# Success: evolutionary and structural properties of amino acids prove effective for succinylation site prediction

**DOI:** 10.1186/s12864-017-4336-8

**Published:** 2018-01-19

**Authors:** Yosvany López, Alok Sharma, Abdollah Dehzangi, Sunil Pranit Lal, Ghazaleh Taherzadeh, Abdul Sattar, Tatsuhiko Tsunoda

**Affiliations:** 1 Department of Medical Science Mathematics, Medical Research Institute, Tokyo Medical and Dental University, Tokyo, Japan; 2 Laboratory for Medical Science Mathematics, RIKEN Center for Integrative Medical Sciences, Yokohama, Kanagawa, Japan; 3 Institute for Integrated and Intelligent Systems, Griffith University, Brisbane, Australia; 4 School of Engineering & Physics, University of the South Pacific, Suva, Fiji; 5 Department of Computer Science, School of Computer, Mathematical, and Natural Sciences, Morgan State University, Baltimore, Maryland, USA; 6 School of Engineering & Advanced Technology, Massey University, Palmerston North, New Zealand; 7 School of Information and Communication Technology, Griffith University, Brisbane, Australia; 8 CREST, JST, Tokyo, 113-8510 Japan

**Keywords:** Post-translational modification, Lysine succinylation, Protein sequences, Amino acids, Prediction

## Abstract

**Background:**

Post-translational modification is considered an important biological mechanism with critical impact on the diversification of the proteome. Although a long list of such modifications has been studied, succinylation of lysine residues has recently attracted the interest of the scientific community. The experimental detection of succinylation sites is an expensive process, which consumes a lot of time and resources. Therefore, computational predictors of this covalent modification have emerged as a last resort to tackling lysine succinylation.

**Results:**

In this paper, we propose a novel computational predictor called ‘Success’, which efficiently uses the structural and evolutionary information of amino acids for predicting succinylation sites. To do this, each lysine was described as a vector that combined the above information of surrounding amino acids. We then designed a support vector machine with a radial basis function kernel for discriminating between succinylated and non-succinylated residues. We finally compared the Success predictor with three state-of-the-art predictors in the literature. As a result, our proposed predictor showed a significant improvement over the compared predictors in statistical metrics, such as sensitivity (0.866), accuracy (0.838) and Matthews correlation coefficient (0.677) on a benchmark dataset.

**Conclusions:**

The proposed predictor effectively uses the structural and evolutionary information of the amino acids surrounding a lysine. The bigram feature extraction approach, while retaining the same number of features, facilitates a better description of lysines. A support vector machine with a radial basis function kernel was used to discriminate between modified and unmodified lysines. The aforementioned aspects make the Success predictor outperform three state-of-the-art predictors in succinylation detection.

**Electronic supplementary material:**

The online version of this article (10.1186/s12864-017-4336-8) contains supplementary material, which is available to authorized users.

## Background

Once proteins are translated in the ribosome they undergo a series of chemical modifications known as post-translational modifications (PTMs). These PTMs play multiple biological roles, which influence cellular functions through complex post-translational networks [1, 2], by adding functional groups to specific residues in a protein. Among such PTMs are methylation [3], ubiquitination [4], acetylation [5] and phosphorylation [6]. Although a great number of PTMs have been extensively studied, a new modification coined succinylation [7, 8], has recently caught the interest of the research community. Succinylation was identified through mass spectrometry and sequence alignment [9], and reportedly contributes to the structure and function of proteins [8]. Succinylated enzymes are known to have essential roles in mitochondrial and fatty acid metabolism [10], whereas modified histones were shown to influence the function of chromatin [11].

Understanding how the succinylation mechanism works is of vital importance because of the involvement of this biological mark in cellular processes. Nevertheless, the detection of succinylation sites by traditional experimental techniques has proven to be expensive and time-consuming. In order to overcome these downsides, computational methods have emerged as a necessary detection approach. Some lysine succinylation predictors, which make use of the amino acid composition of proteins, are: iSuc-PseAAC which employs the position-specific propensity of peptides and the pseudo amino acid composition in order to train a support vector machine [12], iSuc-PseOpt [13] and pSuc-Lys [14] that incorporate the sequence-coupling effects into the composition of amino acids and include the k-nearest neighbor strategy for dealing with class imbalance (for prediction purposes, iSuc-PseOpt uses a random forest algorithm [13] whereas pSuc-Lys utilizes an ensemble of random forest classifiers [14]), SucPred which is a learning algorithm that only regards positive and unlabeled samples [15], SuccinSite that incorporates encoding schemes such as k-spaced amino acid pairs, binary scoring and amino acid index properties as input to a random forest classifier [16], and SuccFind which employs evolutionary information along with an improved feature strategy for optimization [17].

However, none of the above methods made use of a combination of structural and evolutionary information. The critical consideration of evolutionary features has been highlighted by a former study, which identified homologous succinylated proteins and conserved orthologs for them in several species [18]. In spite of all the efforts so far, the accurate detection of succinylated residues remains extremely limited. Therefore, new approaches, able to accurately discriminate between succinylated and non-succinylated lysine residues, are absolutely necessary. In this paper, we propose a novel computational predictor called ‘Success’, which efficiently uses structural features such as accessible surface area (ASA), backbone torsion angles and local structure conformations in addition to evolutionary information from the position-specific scoring matrix (PSSM) of proteins for predicting succinylation sites. We regarded the above characteristics due to their reportedly significance for lysine succinylation prediction. For instance, ASA has been previously employed to determine the surface density of succinylated amino groups in pharmacokinetic analyses [19], whereas lysine succinylation was reported to be evolutionarily conserved [8].

This study used a collection of 670 proteins, which contained annotated succinylated and non-succinylated lysines, from two PTM databases [20, 21]. When these sites were retrieved they amounted to 1782 and 18,344 succinylation and non-succinylation residues, respectively. For each lysine residue, we retrieved the sequence stretch comprising 15 amino acids upstream and downstream of it for feature extraction. The PSSM and the local structure with the highest probability were computed for each protein sequence. Both features were used for extracting the submatrix corresponding to each peptide sequence (15 residues, lysine residue, 15 residues) and subsequently converted to bigram probabilities [22]. Considering all the structural and evolutionary features, each lysine residue was defined by a 657-component vector. Because the numbers of succinylation and non-succinylation sites were hugely disproportionate, the resulting training matrix turned out to be unbalanced. For ameliorating this imbalance we employed a k-nearest neighbor strategy [13]. Subsequently, the remaining non-redundant sites were employed to train a support vector machine with a radial basis function kernel for prediction. We compared ‘Success’ with three benchmark predictors (iSuc-PseOpt [13], pSuc-Lys [14] and SuccinSite [16]). As a result, ‘Success’ showed a significant improvement in performance, being able to accurately predict succinylated lysines with 0.866 sensitivity, 0.838 accuracy and 0.677 Matthews correlation coefficient. To the best of our knowledge, these results have not been attained by any available predictor.

## Methods

The proposed predictor combines structural and evolutionary information of amino acids with bigram profiles [22] for detecting succinylation and non-succinylation sites. The following sections describe the protein sequence dataset, the computed features, and the support vector machine employed for prediction.

### Protein dataset

This study regarded a collection of 670 protein sequences, which were obtained from two PTM databases [20, 21]. The lysine residues of each protein sequence were previously annotated as succinylated or non-succinylated. Because of this, every sequence was analysed and its succinylation and non-succinylation residues were retrieved. As a result, 1782 positive lysines (succinylated) and 18,344 negative lysines (non-succinylated) were obtained.

### Structural and evolutionary features

Each protein sequence was used for computing ten different characteristics related to ASA, backbone torsion angles, secondary structure and position-specific scoring matrix (PSSM). The first three types of characteristics were calculated by SPIDER2 [23], a newly developed tool that has reportedly provided reasonable outcomes when it comes to predicting the structural features of proteins [24–29]. For the computation of the PSSM, we used the PSI-BLAST program [30]. These characteristics are detailed in the following subsections.

### Accessible surface area

ASA shows the approximate accessible area of each amino acid to a particular solvent in the 3D configuration of a protein [31, 32]. Because the ASA value of an amino acid depends on the protein configuration, considering the predicted values tends to be more informative than using an experimentally determined general value. ASA was computed for proteins with known 3D structures by the SPIDER2 tool [23]. As a result, for each amino acid in a protein sequence, we obtained one numeric ASA value.

### Secondary structure

Secondary configuration provides useful information to understand the local 3D structure of proteins. This structure can be studied by looking at the amino acid contributions to local protein structures, namely, helix (*ph*), strand (*pe*) and coil (*pc*). Therefore, we run the SPIDER2 tool [23] on each protein sequence and predicted the contribution likelihood of every amino acid to the three aforementioned local structures. For each protein, we obtained three numerical vectors, each representing a different local structure. In addition, SPIDER2 returns the local structure with the highest likelihood as an *L* × 3 matrix, where *L* indicates the length of the protein sequence and the three columns are the contribution likelihoods to the three local structures (helix, strand and coil). Hereafter, this matrix will be referred to as *SSpre*.

### Backbone torsion angles

While secondary structure regards the local configuration of amino acids of a protein [27], torsion angles complement the secondary structure feature by providing continuous information about the local structure of proteins. For instance, the backbone torsion angles ϕ and Ψ provide continuous information about the interaction of local amino acids along the protein backbone [33, 34]. More recent works have proposed two new angles based on the dihedral angles θ and τ [26]. To take the four angles into consideration, we run SPIDER2 [23] on each protein sequence, and attained four numerical vectors referred to as ϕ, Ψ, θ and τ hereafter.

### Position-specific scoring matrix

The PSSM has been shown to provide useful evolutionary information about proteins [23–25, 35–37]. This matrix contains the substitution probability of each amino acid in a protein with all the amino acids of the genetic code. In order to compute such probabilities, we aligned each protein sequence to those in Protein Data Bank [38] with the PSI-BLAST algorithm [30]. PSI-BLAST was run on non-redundant proteins, with a threshold of 0.001 and three iterations. For each protein in our benchmark dataset, we thus obtained its respective PSSM which consisted of the linear probabilities of amino acids. The resulting PSSM will have a size of *L* × 20, where *L* is the protein length and the 20 columns represent the amino acids of the genetic code.

### Lysine residues as feature vectors

**Fig. 1:**
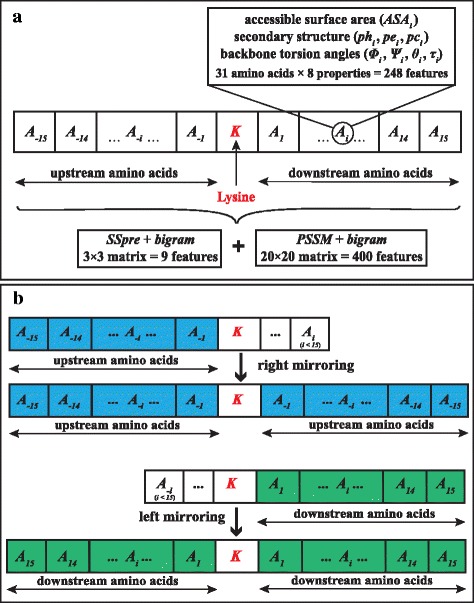
Description of a lysine residue (**a**) with enough amino acids to both sides, and (**b**) with missing upstream (left) and downstream (right) amino acids

Each lysine residue was described in terms of its 15 upstream and 15 downstream amino acids (Fig. 1a). The optimal residue window around a lysine has been widely explored [13, 39, 40]. Previous studies regarded different window sizes and concluded that the 15 amino acids upstream and downstream of a lysine provide useful information about succinylation sites. For this specific study, we also considered several residue windows and trained the predictor (see Additional file 1). Consequently, the same conclusions were drawn. In cases where a lysine was positioned close to either protein terminus, the gap of 15 (upstream or downstream) amino acids was filled by the mirror effect of amino acids [13] (Fig. 1b).

Now let us consider the following peptide *P*,

1 $$ P=\left\{{A}_{-15},{A}_{-14},,\dots, {A}_{-2},{A}_{-1},K,{A}_1,{A}_2,\dots, {A}_{14},{A}_{15}\right\} $$

which describes the lysine *K*, and comprises *A*_−*i*_ (1 ≤ *i* ≤ 15) upstream and *A*_*i*_ (1 ≤ *i* ≤ 15) downstream amino acids. Thereby, each succinylated or non-succinylated lysine was represented by a peptide *P* consisting of 31 amino acids (including itself). The 31 amino acids are thus expressed by structural characteristics, such as *ASA*, *ph*, *pe*, *pc*, *ϕ*, *Ψ*, *θ* and *τ*. The evolutionary features of peptide *P* were represented by bigram profiles [22], extracted from the PSSM and denoted here as *PSSM* + *bigram*. Similarly, the secondary structure with the highest likelihood was also represented in terms of bigram profiles, extracted from *SSpre* and denoted as *SSpre* + *bigram*. The transformation *PSSM* + *bigram* returns a 20 × 20 matrix (or a 400-dimensional feature vector), whereas that of *SSpre* + *bigram* results in a 3 × 3 matrix (or a 9-dimensional feature vector). These two vectors were then used to obtain the corresponding information about each lysine in peptide *P*. We employed the bigram feature extraction technique because of its promising results in solving protein analysis problems [22, 41–48]. The bigram approach is independent of window sizes, which has advantage in this particular study. In other words, it returns 400- (for *PSSM*) and 9- (for *SSpre*) dimensional feature vectors regardless of the residue window size. Previous studies have shown that using a large residue window could provide necessary information for discriminating between lysines and their neighbouring amino acids [13]. Therefore, the bigram approach enables us to resize the residue window around a lysine without necessarily increasing the number of features.

The features *PSSM* and *SSpre* were transformed into bigram profiles as described below. Let a PSSM of size *L* × 20 be *P*_*s*_ whose elements *m*_*pq*_ represent the transitional probabilities of *q*-th amino acids at *p*-th locations in the protein sequence. Thereby, matrix *P*_*s*_ would be represented by a bigram profile [22] as

2 $$ {B}_{p,q}=\sum \limits_{k=1}^{30}{m}_{k,p}{m}_{k+1,q} $$

where 1 ≤ *p* ≤ 20 and 1 ≤ *q* ≤ 20.

Eq. (2) will consequently return a 20 × 20 bigram occurrence matrix *B*, which consists of all the bigram frequencies *B*_*p*, *q*_ (for *p* = 1, 2, …, 20 and *q* = 1, 2, …, 20). This bigram matrix *B* (or *PSSM* + *bigram*) was transformed into a feature vector as

3 $$ F={\left[{B}_{11},\dots, {B}_{ij},\dots, {B}_{20,20}\right]}^T $$

for *i* = 1, …, 20 and *j* = 1, …, 20, and where superscript *T* denotes transpose.

In a similar way, the bigram matrix *B*^′^ for *SSpre* (or *SSpre* + *bigram*) can be described as

4 $$ B{\hbox{'}}_{p,q}=\sum \limits_{k=1}^{30}{r}_{k,p}{r}_{k+1,q} $$

where 1 ≤ *p* ≤ 3 and 1 ≤ *q* ≤ 3, and where elements *r*_*p*, *q*_ are the transition probabilities of each amino acid to the three local conformations (helix, strand and coil).

The bigram matrix *B*^′^ was also transformed into a feature vector as

5 $$ {F}^{\hbox{'}}={\left[B{\hbox{'}}_{11},\dots, B{\hbox{'}}_{ij},\dots, B{\hbox{'}}_{3,3}\right]}^T $$

for *i* = 1, …, 3 and *j* = 1, …, 3.

### Support vector machine for classification

Support vector machine (SVM) is a well-known pattern classification scheme [49], which has been successfully used in regression and classification applications [50–55]. The ultimate goal of SVMs is to maximize the margin between hyperplanes, which represent linear boundaries between classes. To deal with non-linear boundaries, function kernels were consequently introduced [56]. These functions could be radial basis, polynomial or linear. In this work, we designed a SVM that makes use of a radial basis function kernel to find a margin between succinylated (*y*_*i*_ =  + 1) and non-succinylated (*y*_*i*_ =  − 1) lysine residues. If the feature vector of *i*-th lysine residue is defined as *x*_*i*_ with class label *y*_*i*_ (either succinylated or non-succinylated), then an unknown lysine residue *x*^′^ can be predicted by the following function,

6 $$ {y}^{\hbox{'}}=\operatorname{sign}\left(\sum \limits_{i=1}^n{\alpha}_i{y}_iK\left({x}_i,{x}^{\hbox{'}}\right)+\beta \right) $$

where *α*_*i*_ are adjustable weights, *β* represents a bias, *n* is the number of samples and *K*() indicates the radial basis function kernel. The SVM classifier was designed with the Weka tool (*C* = 1, tolerance = 0.001, ϵ = 10^−12^ and γ = 0.01) [57].

## Results and discussion

Any computational approach, aimed at predicting succinylation sites, requires a critical assessment of its performance. The following sections explain the statistical metrics used for evaluation purposes as well as the comparison of the Success predictor and three state-of-the-art predictors.

### Performance metrics

We have considered four well-defined metrics for assessing the performance of the Success predictor and other recently proposed predictors. These metrics include sensitivity, specificity, accuracy and Matthews correlation coefficient (MCC) [41, 58–62]. Sensitivity, which varies between 0 and 1, evaluates the correctness of succinylation site identification. A value of 0 indicates the inability of the predictor to detect succinylated lysines (true positive rate), whereas that of 1 depicts a predictor able to correctly identify all the succinylated lysines. Specificity assesses the capability of a predictor to recognize non-succinylation sites (true negative rate). It varies between 0 (completely incorrect classification) and 1 (completely correct classification). Accuracy measures the total number of correctly classified lysine residues, and ranges from 0 (the least accurate predictor) to 1 (the most accurate predictor). MCC indicates the classification quality of a predictor. A value of −1 indicates a completely negative correlation, whereas that of +1 means a highly positive correlation.

Now let us consider a benchmark dataset, which consists of *K*^+^ succinylated sites and *K*^−^ non-succinylated sites. This can be further expressed as

7 $$ {K}^{+}={\mathrm{K}}_{+}^{+}+{\mathrm{K}}_{-}^{+} $$

8 $$ {K}^{-}={\mathrm{K}}_{-}^{-}+{\mathrm{K}}_{+}^{-} $$

where ${\mathrm{K}}_{+}^{+}$ and ${\mathrm{K}}_{-}^{+}$ are the succinylated residues correctly classified (true positives) as such, and incorrectly classified as non-succinylated sites (false negatives), respectively. Likewise, ${\mathrm{K}}_{-}^{-}$ and ${\mathrm{K}}_{+}^{-}$ are those non-succinylated sites correctly classified (true negatives) as such, and incorrectly classified as succinylated sites (false positives), respectively. The above statistical metrics could be defined as

9 $$ Sensitivity=\frac{K_{+}^{+}}{K^{+}} $$

10 $$ Specificity=\frac{K_{-}^{-}}{K^{-}} $$

11 $$ Accuracy=\frac{K_{+}^{+}+{K}_{-}^{-}}{K^{+}+{K}^{-}} $$

12 $$ MCC=\frac{\left({K}_{-}^{-}\times {K}_{+}^{+}\right)-\left({K}_{+}^{-}\times {K}_{-}^{+}\right)}{\sqrt{\left({K}_{+}^{+}+{K}_{-}^{+}\right)\left({K}_{+}^{+}+{K}_{+}^{-}\right)\left({K}_{-}^{-}+{K}_{-}^{+}\right)\left({K}_{-}^{-}+{K}_{+}^{-}\right)}} $$

Any method that performs the highest in all of these metrics would be the ideal predictor. However, an improved predictor should at least show a higher sensitivity when compared with other approaches. This is because a predictor with lower sensitivity is unable to correctly detect succinylated lysine residues, and hence inappropriate for tackling such prediction problems.

### Cross-validation strategy

In order to accurately assess the performance of the Success predictor in each statistical metric, we utilized a cross-validation procedure. Two commonly used cross-validation approaches include n-fold cross-validation and jackknife [63, 64]. Although jackknife is regarded to be the least arbitrary approach and yields unique outcomes for a dataset [65], we implemented the n-fold cross-validation procedure which requires less processing time. This cross-validation strategy was conducted in five steps as follows:

*Step 1*. Randomly partition the initial dataset into *n* parts of roughly equal size.

*Step 2.* Retain *n* − 1 folds as training data and the remaining fold as validation data.

*Step 3.* Use the training samples for estimating the predictor parameters.

*Step 4.* Compute the four statistical metrics on the validation fold.

*Step 5.* Repeat Step 1 to Step 4 *n* times and calculate the average of each metric.

In this study, we carried out 6-, 8- and 10-fold cross-validations whose results are presented in the subsequent sections.

### Dataset balancing

Our benchmark dataset comprised 670 protein sequences, which accounted for 1782 succinylation sites (positive set) and 18,344 non-succinylation sites (negative set). Such a difference between positive and negative sets indicates a huge imbalance between both classes. Although it is reasonable to assume that the number of non-succinylated lysines might be greater than that of succinylated lysines, this disproportion can severely bias any machine learning classifier.

A long list of methods, aimed at dealing with class imbalance, have been proposed. For instance, sampling methods balance the class distribution by subsampling the majority class, or by sampling with replacement the minority class [66]; ensemble methods consider the majority class in a supervised manner, or learn the characteristics of the original majority class in an unsupervised manner [67]; other methods remove noise and instances in the boundaries [68], or remove instances far away from the decision boundary from the majority class [69]. However, because the k-nearest neighbor strategy has been widely used in this scenario [12, 13], we also implemented it in order to establish a fair comparison with previous predictors. In doing so, we first considered a *k* = 10 (derived from a data ratio of 10:1), and eliminated any negative sample whose 10-nearest neighbours included at least one positive sample. We subsequently increased the *k* value until similar numbers of positive and negative samples were obtained. As a result, the number of negative samples was drastically reduced to 1872 samples. Both sets were then used to perform n-fold cross-validation, and assess the Success predictor against three benchmark predictors [13, 14, 16].

### Success versus benchmark predictors

We compared the Success predictor with three recently proposed predictors, namely, iSuc-PseOpt [13], SuccinSite [16] and pSuc-Lys [14]. These predictors are available as active web servers to which any protein sequence can be uploaded for succinylation site identification. It is worth noting that many of our query proteins were utilized to train these predictors, and therefore the results could be somehow biased in their favour. Besides the performance of the three approaches (iSuc-PseOpt [13], SuccinSite [16] and pSuc-Lys [14]) was reported on the validation data (i.e., samples held-out for testing during n-fold cross-validation). For the Success predictor, this validation data was not used to estimate its training parameters, and thereby we could easily provide the resulting AUCs (area under the curve) for 6-, 8- and 10-fold cross-validations. However, because it was unknown which samples the three benchmark predictors used for training we were unable to report their respective AUCs.

The performance of all the predictors is summarized in Table 1. It can be clearly observed that the proposed predictor outperforms all the benchmark predictors in metrics such as sensitivity, accuracy and MCC. For instance, sensitivity was significantly improved by 40.8%, accuracy by 15%, and MCC by 43.7%. To the best of our knowledge, these promising results have not been achieved by any predictor in the literature. Although SuccinSite [16] showed a high specificity (0.902), its sensitivity was very poor (~0.3), which indicates that ~70% of succinylated lysine residues remained undetected. In addition, the Success predictor reached AUCs of 0.838 for 6-, 8- and 10-fold cross-validations. One of the reasons why the specificity of the proposed predictor turned out lower than that of benchmark methods is because of the extensive removal of negative instances. Those removed sites, though close to positive sites, appear to contain useful information. Nevertheless, this strategy proves to significantly improve sensitivity.

**Table 1:** Performance of the Success predictor and three benchmark predictors

Predictor	Sensitivity	Specificity	Accuracy	MCC
iSuc-PseOpt [13]	0.615	0.779	0.699	0.400
SuccinSite [16]	0.302	**0.902**	0.609	0.256
pSuc-Lys [14]	0.587	0.864	0.729	0.471
Success (6-fold cross-validation)	0.861	0.815	**0.838**	**0.677**
Success (8-fold cross-validation)	**0.866**	0.809	0.837	0.676
Success (10-fold cross-validation)	0.864	0.811	0.837	0.676

The highest values are highlighted in bold

The above results clearly illustrate the capability of the Success predictor to accurately discriminate between succinylation and non-succinylation sites. Such a combination of evolutionary and structural information apparently provides accurate descriptions of succinylated lysines. Additionally, the transformation of *PSSM* and *SSpre* matrices by the bigram feature extraction technique contributes to effectively refine the information of the surrounding amino acids, and thereby capture the differences between each type of lysine. In order to substantiate the previous claim about the importance of the bigram approach, we also trained the proposed predictor without considering any bigram transformation. However, the highest statistical metrics were achieved when the bigram approach was taken into consideration (Table 2). Finally, the SVM classifier with a radial basis function kernel appears to find a maximal hyperplane separation when evolutionary and structural characteristics are employed.

**Table 2:** Performance of the Success predictor without regarding the bigram feature extraction strategy

	cross-validation		
	6-fold	8-fold	10-fold
Sensitivity	0.860	0.859	0.859
Specificity	0.813	0.813	0.809
Accuracy	0.836	0.835	0.834
MCC	0.673	0.672	0.669
AUC	0.836	0.836	0.834

### Insights into succinylation prediction

We manually analyzed the proteins whose succinylation sites were predicted by the predictors: Success, iSuc-PseOpt [13], SuccinSite [16] and pSuc-Lys [14] (see Additional file 2). It turned out that the four predictors successfully detected all the succinylation sites of specific proteins. These proteins included succinate-CoA ligase subunit alpha (UniProtKB ID Q9WUM5) whose absence causes severe disorders with antenatal manifestations [70], serine hydroxymethyltransferase (UniProtKB ID B1XB26) which regulates the metabolic partitioning of methylenetetrahydrofolate [71], and glutamate dehydrogenase 1 (UniProtKB ID P00366) which is involved in the breakdown and synthesis of the neurotransmitter glutamate [72]. However, the Success predictor was the only one capable of detecting all the succinylation sites of proteins involved in apoptosis and cytoskeleton functions. Some of these proteins included elongation factor 1-alpha 1 (UniProtKB ID P10126) which regulates apoptosis and actin cytoskeleton, and acts as a mediator of lipotoxicity [73] as well as T-complex protein 1 subunit gamma (UniProtKB ID E9Q133) which contributes to assemble and fold cytoskeleton proteins [74]. In addition, the Success predictor correctly detected the only succinylation site, which went undetected by the three benchmark predictors, of other proteins. Two of these proteins are transketolase (UniProtKB ID P40142) that affects the NAPDH production in order to counteract oxidative stress [75], and RNA polymerase I-specific transcription initiation factor RRN3 (UniProtKB ID B2RS91) which acts as a connector between RNA polymerase I and transcription factors [76]. Nevertheless, such a unique succinylated lysine was only detected by iSuc-PseOpt [13], SuccinSite [16] and pSuc-Lys [14] predictors for proteins such as galectin (UniProtKB ID B1AQR8), which acts as an immunomodulatory and enhances transforming growth factor-b signaling [77]. For other proteins, their only succinylated lysine was discovered by all the predictors. These proteins included peptidyl-prolyl *cis*-*trans* isomerase FKBP3 (UniProtKB ID Q62446) which stimulates auto-ubiquitylation and proteasomal degradation [78], proline dehydrogenase (UniProtKB ID F6YFQ5) that causes DNA damage-induced senescence [79], and sulfite oxidase (UniProtKB ID Q8R086) which catalyses the oxidation of toxic sulfite to sulfate [80]. Finally, none of the predictors was able to detect the succinylation sites of other proteins. A few examples are lon protease homolog (UniProtKB ID Q8CGK3) which recognises and degrades unfolded proteins [81], caveolin (UniProtKB ID D3Z0J2) which is involved in vesicular transport, cholesterol homeostasis, signal transduction and tumor suppression [82], and kinesin-like protein (UniProtKB ID E9PWU7) that caps microtubules released from the centrosome during interphase [83].

These results indicate that although the Success predictor detected a large number of succinylation sites in comparison to the other predictors, all the predictors should be used in a complementary way for more complete outcomes.

## Conclusions

This study proposes a new computational predictor called ‘Success’, which is aimed at detecting succinylation sites of modified proteins. The proposed method makes an optimum use of the structural and evolutionary information of amino acids around lysine residues. Features such as *PSSM* and *SSpre* were transformed into frequency vectors using the bigram feature extraction approach, which proved effective to describe each type of lysine. The studied characteristics were appropriate for the SVM classifier with a radial basis function kernel to find the maximal separation between modified and unmodified lysine residues.

## Additional files

Additional file 1: Performance of the Success predictor using 6-, 8- and 10-fold cross-validation. (DOCX 148 kb)

Additional file 2: Numbers of succinylation sites detected by each predictor. (XLS 119 kb)

## Abbreviations

ASA: Accessible surface area
AUC: Area under the curve
MCC: Matthews correlation coefficient
PSSM: Position-specific scoring matrix
PTM: Post-translational modification
SVM: Support vector machine
