# Supplementary material for: Success: evolutionary and structural properties of amino acids prove effective for succinylation site prediction
Source: BMC Genomics. 2018 Jan 19;19(Suppl 1):923. doi: 10.1186/s12864-017-4336-8 (PMC5781056; doi:10.1186/s12864-017-4336-8)
Supplement: Supplementary file 1 — Performance of the Success predictor using 6-, 8- and 10-fold cross-validation. (DOCX 148 kb) [file 12864_2017_4336_MOESM1_ESM.docx]

**Performance of the Success predictor using 6-, 8- and 10-fold cross-validation**

**5-residue window**

| **Method** | **Sensitivity** | **Specificity** | **Accuracy** | **MCC** | **AUC** |
| --- | --- | --- | --- | --- | --- |
| Success (6-fold cross-validation) | 0.848 | 0.762 | 0.804 | 0.612 | 0.805 |
| Success (8-fold cross-validation) | 0.842 | 0.757 | 0.799 | 0.601 | 0.800 |
| Success (10-fold cross-validation) | 0.842 | 0.760 | 0.800 | 0.604 | 0.801 |

**9-residue window**

| **Method** | **Sensitivity** | **Specificity** | **Accuracy** | **MCC** | **AUC** |
| --- | --- | --- | --- | --- | --- |
| Success (6-fold cross-validation) | 0.854 | 0.795 | 0.824 | 0.649 | 0.824 |
| Success (8-fold cross-validation) | 0.850 | 0.791 | 0.820 | 0.641 | 0.820 |
| Success (10-fold cross-validation) | 0.851 | 0.791 | 0.820 | 0.643 | 0.821 |

**10-residue window**

| **Method** | **Sensitivity** | **Specificity** | **Accuracy** | **MCC** | **AUC** |
| --- | --- | --- | --- | --- | --- |
| Success (6-fold cross-validation) | 0.856 | 0.796 | 0.825 | 0.653 | 0.826 |
| Success (8-fold cross-validation) | 0.850 | 0.794 | 0.821 | 0.644 | 0.822 |
| Success (10-fold cross-validation) | 0.853 | 0.793 | 0.822 | 0.647 | 0.823 |

**11-residue window**

| **Method** | **Sensitivity** | **Specificity** | **Accuracy** | **MCC** | **AUC** |
| --- | --- | --- | --- | --- | --- |
| Success (6-fold cross-validation) | 0.857 | 0.800 | 0.828 | 0.657 | 0.829 |
| Success (8-fold cross-validation) | 0.852 | 0.801 | 0.826 | 0.653 | 0.827 |
| Success (10-fold cross-validation) | 0.852 | 0.798 | 0.824 | 0.650 | 0.825 |

**13-residue window**

| **Method** | **Sensitivity** | **Specificity** | **Accuracy** | **MCC** | **AUC** |
| --- | --- | --- | --- | --- | --- |
| Success (6-fold cross-validation) | 0.863 | 0.809 | 0.836 | 0.673 | 0.836 |
| Success (8-fold cross-validation) | 0.858 | 0.815 | 0.836 | 0.673 | 0.836 |
| Success (10-fold cross-validation) | 0.860 | 0.810 | 0.834 | 0.670 | 0.835 |

**17-residue window**

| **Method** | **Sensitivity** | **Specificity** | **Accuracy** | **MCC** | **AUC** |
| --- | --- | --- | --- | --- | --- |
| Success (6-fold cross-validation) | 0.861 | 0.808 | 0.834 | 0.669 | 0.834 |
| Success (8-fold cross-validation) | 0.864 | 0.810 | 0.836 | 0.674 | 0.837 |
| Success (10-fold cross-validation) | 0.855 | 0.808 | 0.831 | 0.663 | 0.831 |

**19-residue window**

| **Method** | **Sensitivity** | **Specificity** | **Accuracy** | **MCC** | **AUC** |
| --- | --- | --- | --- | --- | --- |
| Success (6-fold cross-validation) | 0.862 | 0.801 | 0.831 | 0.664 | 0.832 |
| Success (8-fold cross-validation) | 0.865 | 0.807 | 0.835 | 0.672 | 0.836 |
| Success (10-fold cross-validation) | 0.860 | 0.802 | 0.830 | 0.662 | 0.831 |

**20-residue window**

| **Method** | **Sensitivity** | **Specificity** | **Accuracy** | **MCC** | **AUC** |
| --- | --- | --- | --- | --- | --- |
| Success (6-fold cross-validation) | 0.860 | 0.802 | 0.830 | 0.662 | 0.831 |
| Success (8-fold cross-validation) | 0.863 | 0.803 | 0.832 | 0.666 | 0.833 |
| Success (10-fold cross-validation) | 0.857 | 0.799 | 0.827 | 0.656 | 0.828 |
